# Diagnostic value of ultrasonic indicators for assessing acute lung injury severity

**DOI:** 10.1038/s41598-025-99848-2

**Published:** 2025-05-18

**Authors:** Dong Han, Yingying Zhang, Huan Yang, Jingqin Fang, Guanhua Li, Ling Zeng, Hao Tang, Tao Li

**Affiliations:** 1 Department of Ultrasound Diagnosis, Daping Hospital, Army Medical University, Chongqing, China; 2 Department of Weapon Injury Bioeffect Assessment, Daping Hospital, Army Medical University, Chongqing, China; 3 Depatment of Field Medical Equiment, Daping Hospital, Army Medical University, Chongqing, China; 4 Department of Critical Care Medicine, Daping Hospital, Army Medical University, Chongqing, China

**Keywords:** Acute lung injury, Ultrasonic volume assessment indicators, Pulmonary artery VTI, Oleic acid, Diagnostic value, Experimental models of disease, Respiratory distress syndrome

## Abstract

Systemic volume changes during acute lung injury (ALI) are closely related to lung injury severity, disease progression, and treatment methods. Twenty-one goats were divided into control, mild injury, and severe injury groups via oleic acid injection. Carotid ultrasound measured carotid diameter and corrected flow time (FTc), while cardiac ultrasound assessed aortic and pulmonary artery velocity–time integral (VTI). Post-euthanasia at 6 h, lung wet-to-dry (W/D) ratio and pathological scores were analyzed. Statistical trends, correlations between ultrasound parameters and lung injury markers, and diagnostic performance via ROC analysis were evaluated. The severe injury group had significantly higher lung W/D ratios and pathological scores than the mild injury group. Carotid ultrasound showed a progressive decrease in carotid diameter and FTc post-injury, with FTc significantly lower in the severe injury group at 6-h. FTc was negatively correlated with lung W/D ratio and pathological scores. Cardiac ultrasound indicated a decreasing trend in aortic and pulmonary artery VTI post-injury, with pulmonary artery VTI significantly lower in the severe injury group at all times and negatively correlated with lung W/D ratio and pathological scores. ROC analysis showed that pulmonary artery VTI had the highest area under the curve (AUC), with values greater than 0.8 at all time points. The combined use of pulmonary artery VTI and carotid FTc had AUC values greater than 0.85 at all time points, peaking at 6-h (AUC = 0.951). In conclusion, pulmonary artery VTI is an excellent indicator for evaluating ALI severity post-injury, and the combination of pulmonary artery VTI and carotid FTc shows strong diagnostic performance for assessing ALI severity.

## Introduction

Acute lung injury (ALI) is characterized by diffuse interstitial and alveolar edema caused by non-cardiac factors, potentially leading to acute hypoxic respiratory failure or respiratory distress^1–3^. ALI often results from alveolar edema, decreased alveolar surfactant, and increased alveolar exudation, which can cause elevated pulmonary arterial pressure, increased cardiac workload, pulmonary edema, and reduced overall body fluid volume, thereby adversely affecting patient prognosis^4,5^. Therefore, volume assessment in ALI is crucial for predicting patient outcomes.

Ultrasound-based volume assessment parameters (UVAIs), characterized by technical simplicity, radiation-free operation, reproducibility, and real-time data acquisition, have been increasingly utilized to evaluate systemic and pulmonary circulation volume status in patients with ALI and to guide clinical fluid management^4,6^. However, some UVAIs, when used alone, can only reflect localized blood volume status and do not fully represent systemic blood volume^7–9^. Additionally, some UVAIs are difficult to obtain, rely heavily on operator skill, and involve subjective image interpretation. Moreover, there is currently a lack of clinical application and experimental research on the use of UVAIs for assessing injury severity in ALI patients. Whether there are differences in the expression levels of UVAIs among patients with varying degrees of ALI post-injury, whether these indicators can serve as diagnostic tools for assessing the severity of ALI, and whether their combined use can enhance diagnostic performance are questions that require further investigation.

Based on the aforementioned questions, our study aims to measure carotid and cardiac UVAIs and analyze their correlation with the severity of extravascular lung water and pathological lung injury in goats post-ALI. Additionally, we seek to evaluate the diagnostic value of various UVAIs in assessing ALI severity, aiming to offer novel insights and methodologies for volume assessment and injury evaluation in clinical ALI patients.

## Results

### Modeling and vital signs examination results

**Fig. 1:**
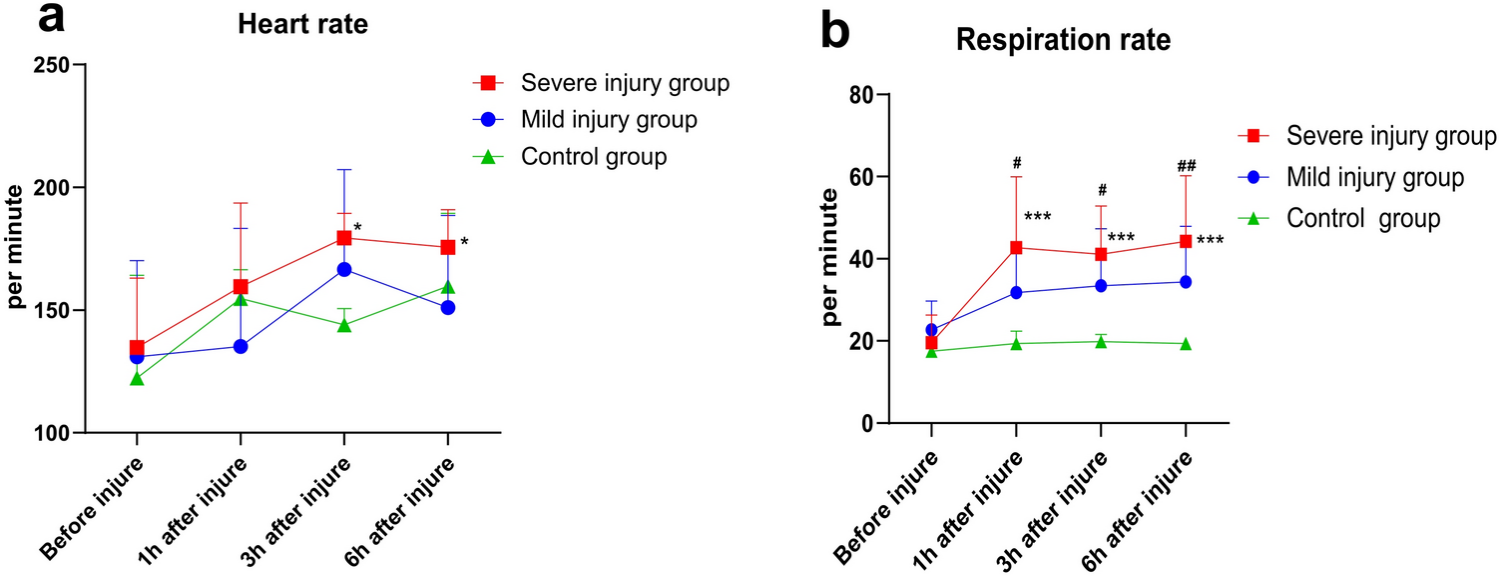
Vital signs of goats. (**a**)–(**b**) Heart rate (**a**) and respiratory rate (**b**) results at pre-injury and 1-, 3-, and 6-h post-injury. *Comparison of post-injury time points with pre-injury within the same group, #Comparison of light or severe injury groups with the control group at the same time point, */# *P* < 0.05, **/## *P* < 0.01, ***/###* P* < 0.001.

All 21 goats were intravenously injected with oleic acid via the auricular vein and underwent various tests at 1-, 3-, and 6-h post-injury. The vital signs results showed that heart rate increased in the post-injury groups at each time point compared to pre-injury. Specifically, there was a statistically significant increase in heart rate at 3- and 6-h post-injury in the severe injury group (all *P* < 0.05, Fig. 1a). Respiratory rate data (Fig. 1b) indicated an increase in respiratory rate post-injury compared to pre-injury levels in the injury groups, with rates lower than those in the control group. Statistically significant differences were observed at 1-, 3-, and 6-h post-injury in the severe injury group and at 1-, 3-, and 6-h post-injury compared to the control group (all *P* < 0.05). These results are consistent with the pathophysiological characteristics of ALI post-injury.

### Pathological examination results

**Fig. 2:**
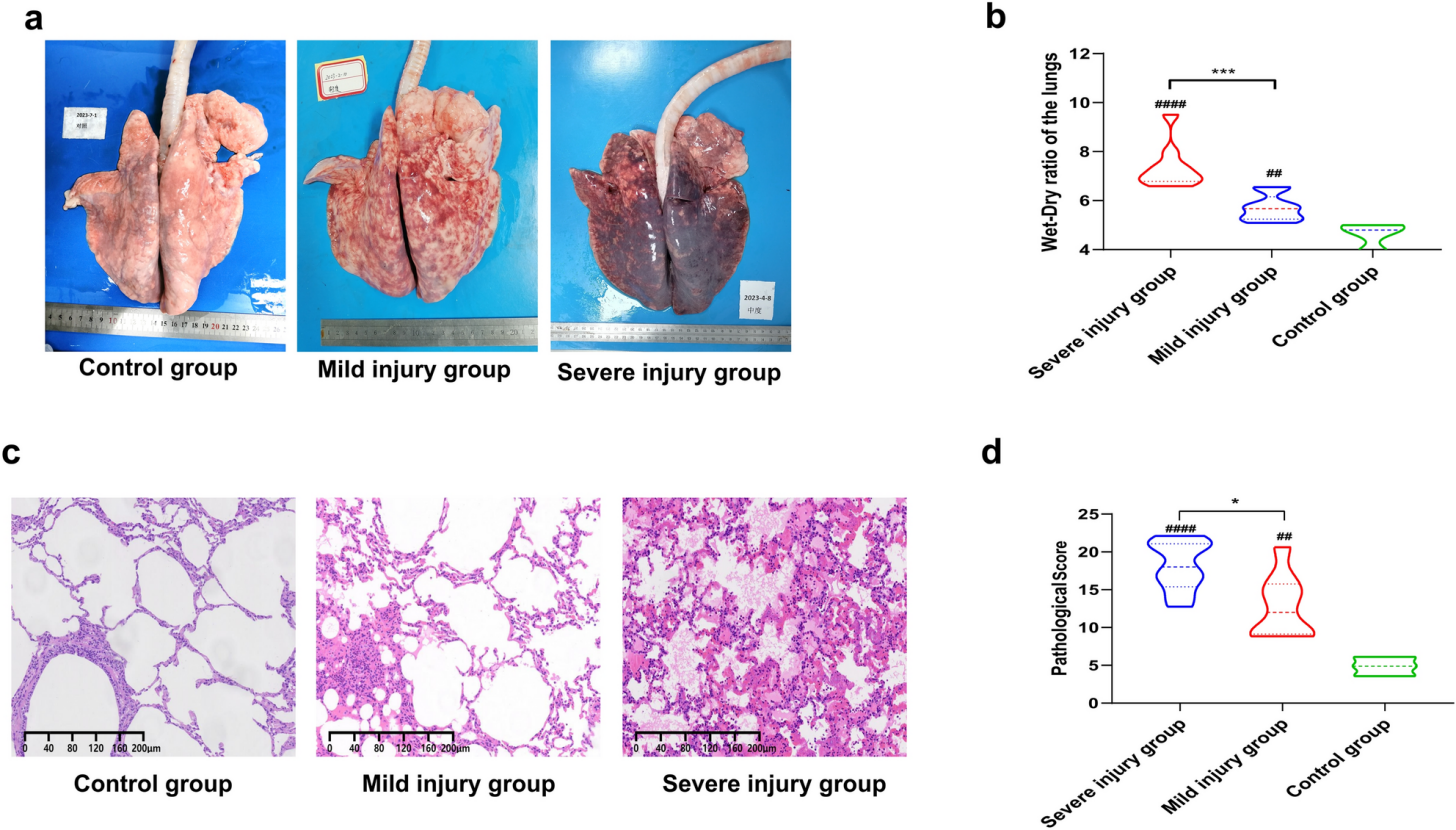
Pathological results of goats. (**a**) Gross pathology results of both lungs in some goats at 6-h post-injury for each group; (**b**) lung wet-to-dry ratio results at 6-h post-injury for each group, *Comparison of light injury group with severe injury group, #Comparison of light or severe injury groups with the control group; (**c**) Histopathological results of some goats at 6-h post-injury (HE ×100); (**d**) pathological injury scores at 6-h post-injury for each group, *Comparison of light injury group with severe injury group, #Comparison of light or severe injury groups with the control group. */# *P* < 0.05, **/## *P* < 0.01, ***/###* P* < 0.001, ****/####* P* < 0.0001.

Macroscopic observation of lung tissues revealed no significant abnormal bleeding in the control group, while varying degrees of damage were observed in the injury groups. The mild injury group mainly exhibited punctate hemorrhages, whereas the severe injury group showed diffuse hemorrhages throughout the lungs (Fig. 2a). Comparison of lung wet-to-dry (W/D) ratios showed that the injury groups had significantly higher ratios than the control group, and the severe injury group had a higher ratio than the mild injury group, with all differences being statistically significant (all *P* < 0.05, Fig. 2b). Histopathological results showed a marked increase in pulmonary hemorrhage areas and significant thickening and congestion of alveolar septa in the severe injury group compared to the mild injury group. Additionally, more neutrophil aggregation was observed in the alveolar septa of the severe injury group, whereas only a small amount of bleeding was seen in the control group (Fig. 2c). Quantitative pathological scoring revealed that both the severe and mild injury groups had significantly higher lung pathological injury scores than the control group, with the severe injury group scoring higher than the mild injury group, all with statistical significance (all *P* < 0.05, Fig. 2d). These results further demonstrate the successful establishment of different severity levels of ALI models in goats induced by oleic acid in this study.

### Carotid artery ultrasound examination results

**Fig. 3:**
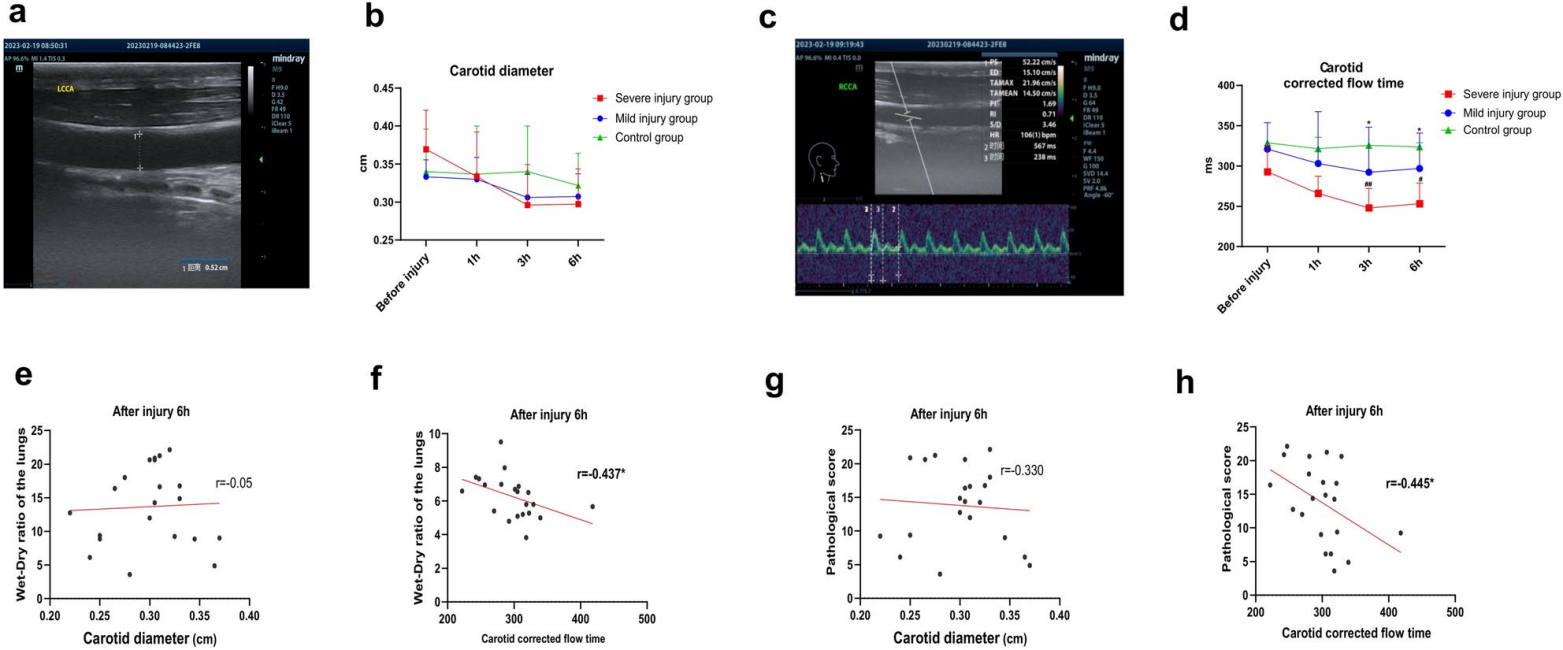
Carotid artery ultrasound results in goats. (**a**) Measurement of carotid artery diameter using two-dimensional ultrasound; (**b**) measurement results of carotid artery diameter at pre-injury and 1-, 3-, and 6-h post-injury, taking the average of both sides; (**c**) measurement of carotid FTc using spectral Doppler ultrasound, time 2: Systolic time; time 3: Cycle time; (**d**) measurement results of carotid FTc at pre-injury and 1-, 3-, and 6-h post-injury, taking the average of both sides; (**e**) and (**f**), Correlation analysis between carotid artery diameter (**e**) and carotid FTc (**f**) with lung wet-to-dry ratio at 6-h post-injury; (**g**) and (**h**), Correlation analysis between carotid artery diameter (**g**) and carotid FTc (**h**) with pathological lung injury scores at 6-h post-injury, *Comparison of light injury group with severe injury group at the same time point, #Comparison of light injury or severe injury groups with the control group at the same time point, */# *P* < 0.05, **/## *P* < 0.01. LCCA: left common carotid artery; RCCA: right common carotid artery; FTc: corrected flow time.

The measurements of carotid artery internal diameter (Fig. 3a,b) showed a decreasing trend in the injury groups post-injury, with values lower than those of the control group; however, these differences were not statistically significant, and there were no significant differences between the injury groups. The results of carotid artery corrected flow time (FTc) (Fig. 3c,d) indicated a gradual decrease in FTc post-injury in the injury groups, with values lower than those of the control group. Specifically, the severe injury group showed statistically significant differences compared to the control group at 3-, and 6-h post-injury (*P* < 0.05), and at 3-, and 6-h post-injury, the FTc of the severe injury group was significantly lower than that of the mild injury group (*P* < 0.05). Subsequently, correlation analysis was conducted between the post-injury 6-h carotid artery internal diameter and FTc with the goats’ lung W/D ratios. The results revealed that there was no statistically significant correlation between the post-injury 6-h carotid artery internal diameter and lung W/D ratio (Fig. 3e), whereas there was a significant negative correlation between carotid artery FTc and lung W/D ratio (*P* < 0.05, Fig. 3f). Finally, correlation analysis was performed between the post-injury 6-h carotid artery internal diameter and FTc with the goats’ lung pathological injury scores. The analysis showed no statistically significant correlation between the post-injury 6-h carotid artery internal diameter and lung pathological injury score (Fig. 3g), while there was a significant negative correlation between carotid artery FTc and lung pathological injury score (*P* < 0.05, Fig. 3h).

### Cardiac ultrasound examination results

**Fig. 4:**
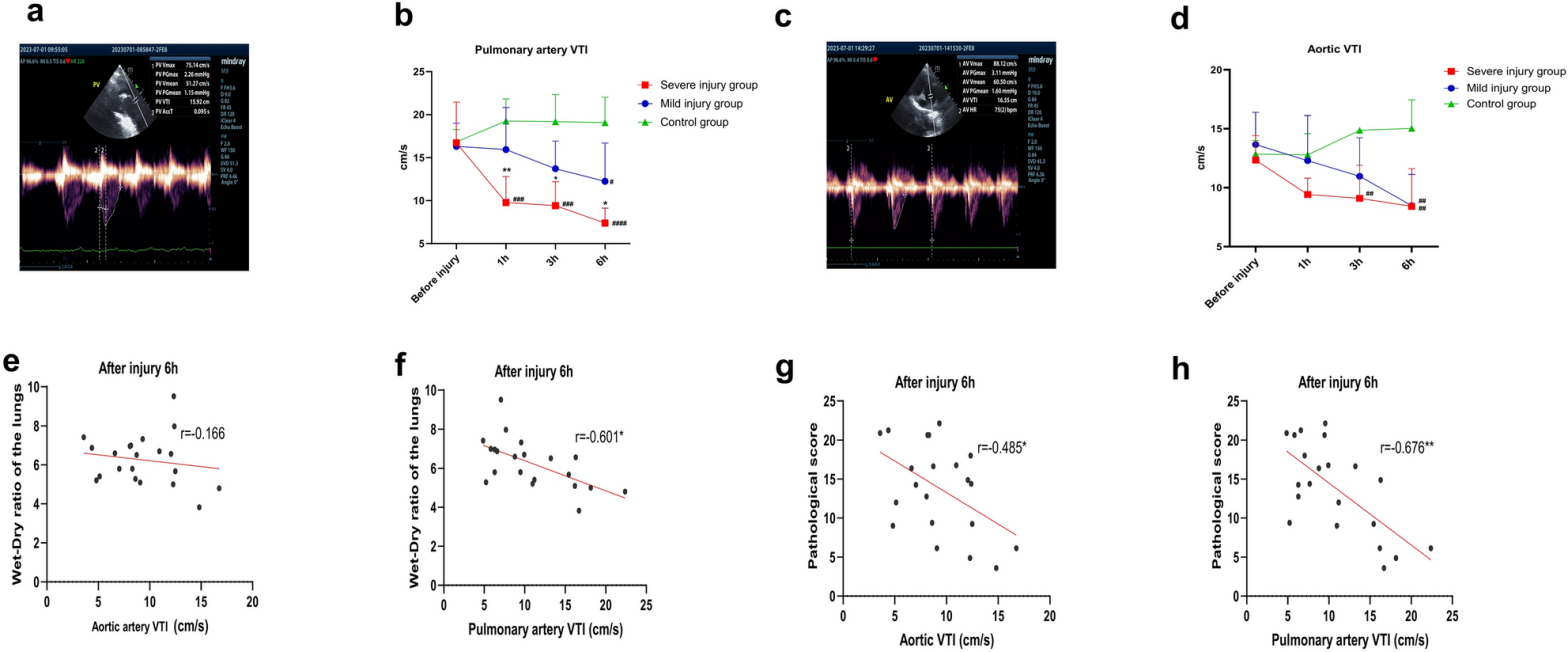
Cardiac ultrasound results in goats. (**a**) Measurement of pulmonary artery VTI using spectral Doppler ultrasound; (**b**) measurement results of pulmonary artery VTI at pre-injury and 1-, 3-, and 6-h post-injury; (**c**) measurement of aortic VTI using spectral Doppler ultrasound; (**d**) measurement results of aortic VTI at pre-injury and 1-, 3-, and 6-h post-injury; (**e**) and (**f**), Correlation analysis between pulmonary artery VTI (**e**) and aortic VTI (**f**) with lung wet-to-dry ratio at 6-h post-injury; (**g**) and (**h**), Correlation analysis between pulmonary artery VTI (**g**) and aortic VTI (**h**) with pathological lung injury scores at 6-h post-injury, *Comparison of light injury group with severe injury group at the same time point, #Comparison of light injury or severe injury groups with the control group at the same time point, */# *P* < 0.05, **/## *P* < 0.01, ***/###* P* < 0.001, ****/####* P* < 0.0001. *PV* pulmonary arterial vascular, *AV* aortic vascular, *VTI* velocity time integral.

The cardiac ultrasound examination results showed a decreasing trend in the post-injury velocity–time integral (VTI) of both the aortic/pulmonary artery in the injury groups. Specifically, the post-injury pulmonary artery VTI in the severe injury group was significantly lower than that in the mild injury group and the control group at each post-injury time point, while the post-injury pulmonary artery VTI in the mild injury group was significantly lower than that in the control group at 6-h post-injury (all *P* < 0.05, Fig. 4a,b). On the other hand, post-injury aortic VTI demonstrated statistically significant reductions compared to controls in the mild injury group at 6 h (*P* < 0.05) and the severe injury group at both 3- and 6-h time points (all *P* < 0.05). Notably, intergroup comparisons revealed no significant VTI differences between mild and severe injury cohorts at any measured intervals (all *P* > 0.05, Fig. 4c,d).

Subsequently, correlation analysis was conducted between the post-injury 6-h aortic/pulmonary artery VTI and the goats’ lung W/D ratios. The results revealed that there was no statistically significant correlation between the post-injury 6-h aortic VTI and lung W/D ratio (*P* > 0.05, Fig. 4e), while the pulmonary artery VTI showed a significant negative correlation with the lung W/D ratio (*P* < 0.05, Fig. 4f).

Finally, correlation analysis was performed between the post-injury 6-h aortic/pulmonary artery VTI and the goats’ lung pathological injury scores. The analysis showed a significant negative correlation between the post-injury 6-h aortic VTI and pulmonary artery VTI and lung pathological injury scores (both *P* < 0.05, Fig. 4g,h).

### Diagnostic performance evaluation

**Fig. 5:**
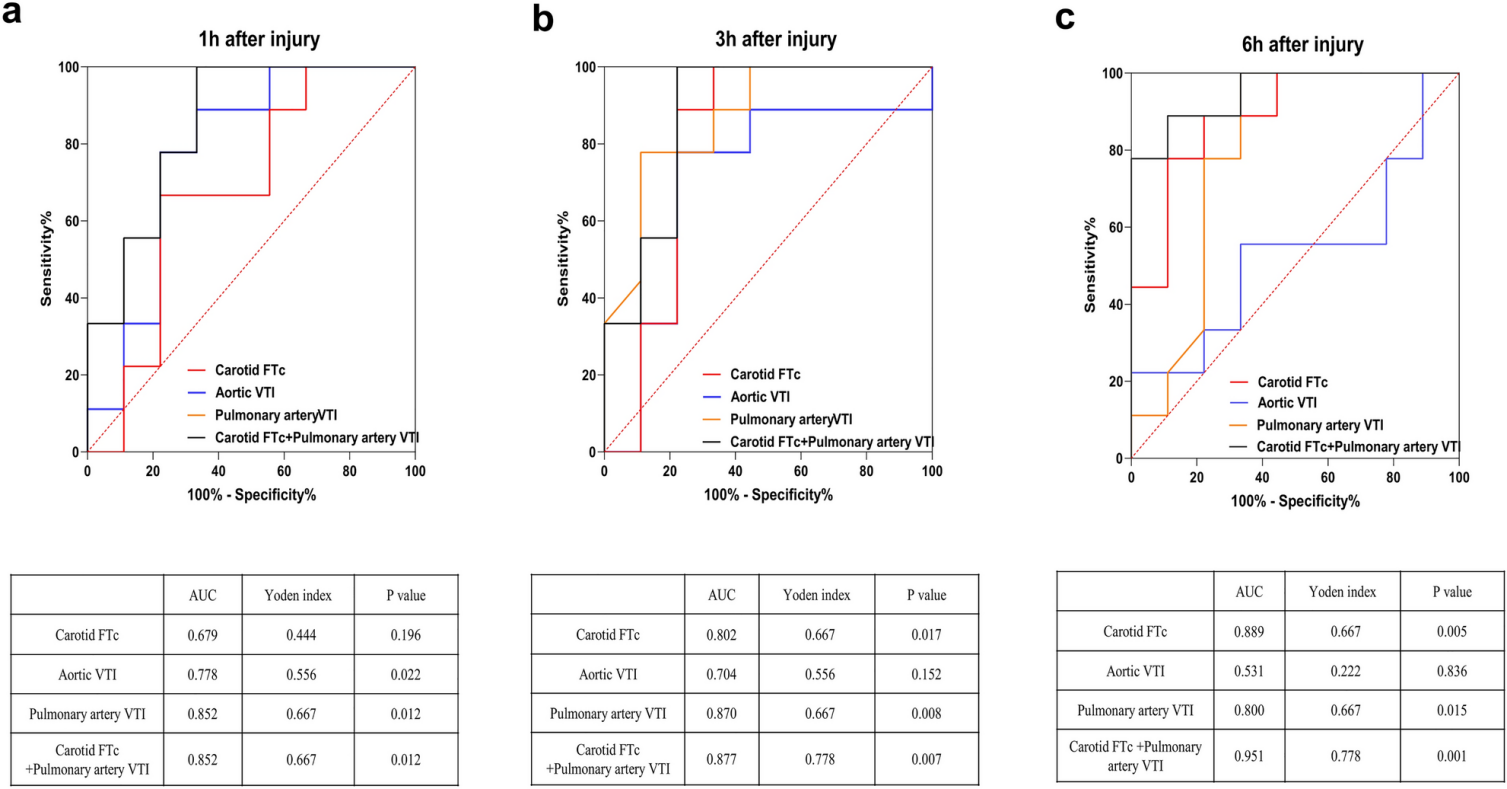
ROC results. ROC results at 1-h (**a**), 3-h (**b**), and 6-h (**c**) post-injury for carotid FTc, aortic VTI, pulmonary artery VTI, and the combination of carotid FTc and pulmonary artery VTI; Red line: carotid FTc; Blue line: aortic VTI; Orange line: pulmonary artery VTI; Black line: combined carotid FTc and pulmonary artery VTI. *ROC* receiver operating characteristic curve, *AUC* area under curve.

The UVAIs with high correlation with lung W/D ratio and lung pathological injury in the previous experiment, including carotid FTc and aortic/pulmonary artery VTI, were selected to plot receiver operating characteristic (ROC) and analyze their diagnostic performance for ALI severity. The results indicated that among these indicators when used alone, pulmonary artery VTI had the highest area under the curve (AUC), with post-injury 1-, 3- and 6-h AUC all > 0.8 (both* P* < 0.05, Fig. 5a–c). Carotid FTc had an AUC > 0.8 at 3- and 6-h post-injury (*P* < 0.05, Fig. 5b,c), while aortic VTI had lower AUC, with AUC < 0.8 at all post-injury time points.

When carotid FTc and pulmonary artery VTI were used in combination, the AUC at all post-injury time points was > 0.85 (all *P* < 0.05), with the highest AUC being 0.951 at 6-h post-injury for the combined use of carotid FTc and pulmonary artery VTI (*P* = 0.001, Fig. 5c). These results suggest that pulmonary artery VTI has significant diagnostic value for assessing ALI severity, and the combined use of pulmonary artery VTI and carotid FTc offers even stronger diagnostic performance.

## Discussion

The pathogenesis of ALI remains incompletely understood, necessitating experimental animal models for mechanistic exploration. Widely used ALI induction methods include lipopolysaccharide, acid aspiration, H_2_O_2_ perfusion, sepsis, and oleic acid^1,10–13^. Oleic acid induces ALI by damaging pulmonary vascular endothelial cells, disrupting surfactant, and triggering a chemical inflammatory response, which mechanically obstructs the pulmonary microvascular bed. This effectively simulates the pathophysiological manifestations of ALI/acute respiratory distress syndrome (ARDS) in patients with severe trauma, multiple fractures, and fat embolism^14–17^. Previous oleic acid-induced lung injury models have primarily used small animals such as rats and rabbits. This study pioneers the construction of an oleic acid-induced ALI model in goats, leveraging their anatomical fidelity to humans (particularly cardiac structure), docile temperament, and accessible vasculature for procedural reliability^18–20^. Post-modeling observations confirmed ALI-associated vital sign changes and pathological hallmarks, with significant histopathological differences (*P* < 0.05) and elevated lung W/D ratios distinguishing injury severity, validating the model’s efficacy.

Research indicates that changes in systemic volume during ALI are closely related to the extent of lung injury, disease progression, and treatment strategies. Earlier studies and clinical trials have shown that volume overload during ALI exacerbates pulmonary edema, damages the alveolar-capillary barrier, and worsens lung injury. Conversely, insufficient volume can lead to inadequate tissue perfusion, organ ischemia, and subsequently, multiple organ dysfunction syndrome^2,21^. Studies have also shown that the volume management strategies for ALI patients vary at different stages of the disease. Early stages may require moderate volume supplementation, while later stages necessitate fluid restriction^1,10^. Thus, timely monitoring of volume changes in ALI patients and appropriate adjustment of fluid management are crucial for improving patient outcomes and prognosis. Currently, clinical indicators used for volume assessment in ALI patients include clinical signs (blood pressure, urine output, skin turgor), hematological tests (hematocrit, hemoglobin concentration), hemodynamic monitoring (central venous pressure, pulmonary artery wedge pressure), and volume responsiveness tests (e.g., blood volume variability indices, pulse pressure variability indices)^7,8^. However, these indicators have limitations: clinical signs can be influenced by other factors and are not highly specific for ALI; hematological tests cannot directly reflect changes in total blood volume; hemodynamic monitoring is invasive and not suitable for all patients; and volume responsiveness tests can be affected by mechanical ventilation and arrhythmias, sometimes failing to accurately reflect true blood volume status^22,23^.

In contrast, ultrasound volume assessment offers several advantages such as being non-invasive, portable, cost-effective, repeatable, and capable of real-time quantification. Previous studies have demonstrated that ultrasound volume indicators, including cardiac output, aortic VTI, pulmonary artery VTI, inferior vena cava collapsibility index, and carotid FTc, can be used to evaluate systemic circulation status, cardiac function, and volume responsiveness in patients with trauma, infection, shock, and ALI. These indicators can effectively guide clinical judgment and treatment^24–27^. However, UVAIs also have certain limitations. Their acquisition relies on the level of operator skill and the quality of the equipment, leading to potential variability. For example, differences in the handling of details such as probe angle, pressure control, and measurement position during image acquisition may lead to significant deviations in the measurement results. Some indicators are influenced by factors such as heart rate, blood pressure, and blood volume, which can reduce their accuracy when used in isolation. Additionally, there is currently a lack of research specifically addressing the use of UVAIs for assessing the severity of ALI.

In this study, based on the anatomical characteristics of goats, we selected four UVAIs: carotid artery diameter, carotid FTc, aortic VTI, and pulmonary artery VTI. The results indicated that pulmonary artery VTI showed a significant decreasing trend post-injury. Its measurements correlated highly with the lung W/D ratio and lung pathological injury scores in goats. The ROC curve analysis showed that pulmonary artery VTI had high AUC values, demonstrating good diagnostic value for assessing ALI severity.

Pulmonary artery VTI primarily measures the ejection velocity from the right ventricle to the pulmonary artery, calculating the VTI over a cardiac cycle. It is used to assess right ventricular ejection capacity, right heart preload, pulmonary hypertension, and pulmonary circulation volume^9,28^. In this study, pulmonary artery VTI could detect differences in blood volume changes among goats with varying degrees of lung injury earlier and with greater specificity than aortic VTI. This may be due to two main reasons: Firstly, in the early stages of ALI, fluid leakage from pulmonary blood vessels into the alveolar interstitial space causes pulmonary edema, reducing pulmonary circulation blood volume. Secondly, the release of inflammatory mediators in ALI increases pulmonary arterial resistance, leading to increased right heart preload. Furthermore, we found a strong correlation between pulmonary artery VTI and both the degree of pulmonary edema and the severity of lung pathological injury in goats post-ALI. This correlation has not been previously reported and may provide a new methodological basis for ultrasound assessment of ALI severity. Additionally, the study found that the combined use of pulmonary artery VTI and carotid FTc significantly enhanced diagnostic performance, offering a potential solution to the issue of insufficient accuracy when using ultrasound volume indicators individually.

This study has several limitations that warrant consideration: the use of goats as experimental subjects introduced ethical and resource constraints, resulting in a small sample size that may compromise statistical power, increase Type II error risks, and limit the representation of biological variability within the target population; anatomical discrepancies, particularly the “pigeon chest” thoracic configuration in goats, precluded the measurement of key echocardiographic parameters (e.g., inferior vena cava diameter), necessitating caution when extrapolating findings to human physiology; furthermore, the UVAIs were correlated solely with extravascular lung water (lung W/D ratio) and histopathological scores, lacking comparisons to established clinical metrics (e.g., central venous pressure), which may introduce interpretive bias. Future research should focus on integrating pulmonary artery VTI and carotid artery FTc with classical clinical indicators (PaO2/FiO2, lung ultrasound scores, serum inflammatory cytokines) through multivariate regression models to enhance diagnostic sensitivity and specificity in ALI patients, while standardizing ultrasound protocols to minimize operator-dependent variability.

In summary, this study successfully established graded ALI models in goats via oleic acid induction, identifying pulmonary artery VTI as the optimal ultrasound volume assessment indicator for severity stratification, with enhanced diagnostic accuracy when combined with carotid FTc. Future directions include validating translational relevance in human cohorts, developing standardized imaging protocols to minimize operator variability, thereby advancing evidence-driven ALI management strategies.

## Methods

### Experimental animal grouping and model construction

Twenty-one male goats weighing between 17 and 27 kg were provided by the Experimental Animal Center of the Army Medical University. Inclusion criteria: general good health and clear ultrasound images of the heart and carotid arteries. Exclusion criteria: presence of underlying diseases, poor general health, and unsatisfactory ultrasound image acquisition (Unable to complete data measurement). The research protocol received approval from the Animal Welfare and Ethical Review Committee of the Army Military Medical University (Approval No. AMUWEC20202140). All animal experimental protocols of the study were conducted following the guidelines outlined in the National Institutes of Health Guide for the Care and Use of Laboratory Animals, and the authors complied with the ARRIVE guidelines. The goats were fasted for 8 h with water deprivation before being weighed. Using a random number method, they were divided into three groups: control group (3 goats), mild injury group (9 goats), and severe injury group (9 goats). Referring to preliminary results from our research group, the modeling conditions were as follows: the control group received a 5 ml physiological saline injection via the auricular vein; the mild injury group received a 0.05 ml/kg oleic acid injection via the auricular vein (Shanghai Meclin Biochemical Technology Co., Ltd.) in three divided doses within 30 min; the severe injury group received a 0.10 ml/kg oleic acid injection via the auricular vein in four divided doses within 30 min. The success criteria for establishing mild and severe ALI models were based on post-mortem lung pathology and lung W/D ratio in goats.

### Vital signs examination

The goats were administered 10 ml of propofol (Xi’an Libang Pharmaceutical Co., Ltd.) via the auricular vein for short-term anesthesia, and an auricular vein cannula was established. The experimental goats were positioned supine and secured on an experimental animal dissection table, with routine skin preparation of the neck, chest, and right lower limb. The goats were placed in a right lateral position, and using the mintti smartho-D2 medical electronic stethoscope (Zhejiang Uniaid Medical Technology Co., Ltd.), heart and lung auscultation areas were identified. Heart rate and respiratory rate were recorded before injury and at 1-, 3-, and 6-h post-injury.

### Pathological examination

After completing all measurements at 6-h post-injury, the goats were euthanized by injecting a 3% pentobarbital sodium solution (100 mg/kg) via the auricular vein. Two intermediate-level pathologists (unaware of the experimental grouping) evaluated the gross lung injury and then extracted one piece each from the upper and lower parts of both lungs (with fixed extraction positions) and placed them in 10% neutral formalin fixative solution (Chongqing Boyi Chemical Reagent Company) for fixation. The specimens were sent to the pathology department of the Army Medical Center for routine dehydration, paraffin embedding, and preparation of 4 nm sections, followed by hematoxylin and eosin (HE) staining. Referring to the method by Mikawa et al.^29^, pathological injury scoring was performed. Specifically, the pathological slides were scanned into the computer using the KF-PRO-005 digital pathology scanning system (Ningbo Jiangfeng Bioinformatics Technology Co., Ltd.) and observed using the K-Viewer software (version 1.7.0.17). Eight random fields were selected for each lung area and observed under 100× and 400× magnification to assess goat lung injuries and assign scores (Table 1). The sum of scores for each indicator under 100× magnification in a field was recorded as the score for that field. The mean of scores from 8 fields was calculated as the pathological injury score for that lung area, and the sum of scores from 4 lung areas was recorded as the overall lung pathological injury score. Lung wet weight was measured, followed by drying in a DHG-9070A blast drying oven (Shanghai Hongdu Electronic Technology Co., Ltd.) at 80 °C for 7 days (12 h per day). Lung dry weight was then measured, and the ratio of lung wet weight to dry weight was calculated as the lung W/D ratio.

**Table 1:** The pathological scoring criteria for lung injury.

Score	0	1	2	3
Alveolar hemorrhage	No hemorrhage	Hemorrhage present, 1–5 alveoli per field with ≤ 5 red blood cells per alveolus	Hemorrhage present, 6–10 alveoli per field with > 5 red blood cells per alveolus	Hemorrhage present, > 10 alveoli per field with > 5 red blood cells per alveolus
Alveolar wall congestion	No congestion	< 1/3 of field with alveolar wall congestion	1/3–2/3 of field with alveolar wall congestion	> 2/3 of field with alveolar wall congestion
Neutrophil aggregation in interstitium	< 5 alveolar septa with neutrophil aggregation per field	5–10 alveolar septa with neutrophil aggregation per field	10–20 alveolar septa with neutrophil aggregation per field	> 20 alveolar septa with neutrophil aggregation per field

### Ultrasonography

The goats were secured to the experimental animal table. For Carotid artery ultrasound examination, the goats were placed in a supine position, while for cardiac ultrasound examination, they were positioned in a left lateral decubitus position.

### Carotid artery ultrasonography

A Mindray M9 portable color Doppler ultrasound diagnostic instrument with an L12-4 s high-frequency linear array probe (9.0 MHz) was used. Before injury and at 1-, 3-, and 6-h post-injury, the goats’ heads were turned approximately 30° to the contralateral side, and the L12-4 s high-frequency linear array probe was used to scan the neck from the neck root to the mandibular angle area, obtaining two-dimensional ultrasound images of the carotid artery in the short axis. The probe was then rotated approximately 90° with the indicator pointing towards the head to obtain long-axis images of the carotid. Pulse Doppler sampling lines were adjusted, and the angle of the sampling line was adjusted to obtain carotid blood flow spectra. The internal diameters of the bilateral carotid were measured at the middle segment of the carotid. The systolic time (ST), cycle time (CT), and corrected flow time (FTc) of the carotid were recorded and calculated using the formula as follows: FTc = ST (ms)/√CT(s). The obtained internal diameters and FTc values were averaged for both sides.

### Cardiac ultrasonography

A Mindray M9 portable color Doppler ultrasound diagnostic instrument with an SP5-1 s phased array probe (3.5 MHz) was used. Before injury and at 1-, 3-, and 6-h post-injury, the SP5-1 s phased array probe was used in the left precordial area intercostal space to scan the heart and great arteries in the short axis, using pulse Doppler for cardiac examination. Blood flow spectra were obtained at the level of the aortic and pulmonary valves, and the VTI of the main and pulmonary arteries was recorded.

### Statistical analysis

Quantitative data were expressed as mean ± standard deviation (SD) if they followed a normal distribution, and non-normally distributed quantitative data were expressed as median (Q1, Q3). Group comparisons were conducted using either unpaired t-tests or two-way analysis of variance (ANOVA) with Sidak’s multiple comparisons test for normally distributed data. For non-normally distributed data, the Mann–Whitney U test was used for intergroup comparisons. Linear regression analysis was employed to assess the correlation between each UVAIs and lung W/D ratio, as well as lung pathological injury scores. Receiver operating characteristic (ROC) analysis was performed to evaluate the UVAIs at different time points for assessing the severity of ALI, and the area under the curve (AUC) was compared using the Z-test. A significance level of *P* < 0.05 was considered statistically significant. GraphPad Prism 8.0 statistical software was used for all statistical analyses.

## Data Availability

The datasets generated during and/or analyzed during the current study are available from the corresponding author on reasonable request.
